# Reduced CARS2 expression elicits a low-grade pro-inflammatory signature in THP-1 macrophages

**DOI:** 10.3389/fimmu.2026.1786365

**Published:** 2026-06-05

**Authors:** Anh-Thu Dang, Paulina Lau, Sébastien Soubeyrand, Ruth McPherson

**Affiliations:** 1 Atherogenomics Laboratory, University of Ottawa Heart Institute, Ottawa, ON, Canada; 2 Division of Cardiology, Ruddy Canadian Cardiovascular Genetics Centre, University of Ottawa Heart Institute, Ottawa, ON, Canada

**Keywords:** CARS2, inflammation, macrophage - cell, mitochondria, THP-1, transcription analysis

## Abstract

Genome-wide association studies have linked *CARS2* locus, encoding the mitochondrial cysteinyl aminoacyl-tRNA synthetase CARS2, to coronary artery disease. Moreover, we previously demonstrated that *CARS2* suppression was associated with a pro-inflammatory expression profile in cell models. However, the mechanisms were largely unexplored. Here, we further examine the changes associated with *CARS2* suppression through bioinformatic and experimental approaches. Analysis of transcriptomic data from *CARS2-*suppressed cells revealed the prominent establishment of an interferon-like response in unpolarized THP-1 macrophages, as well as possible NF-ΚB activation. This was not accompanied by significant activation of STAT1 or NF-ΚB phosphorylation, indicative of interferon pathway activation and NF-ΚB activity, respectively. Rather, an NF-ΚB activity reporter THP-1 model revealed that NF-ΚB activation was modestly diminished in *CARS2*-suppressed cells. Furthermore, suppression of *CARS2*, or a *YARS2* control, similarly reduced radical oxygen species levels without affecting mitochondrial content or membrane potential. However, *YARS2* suppression was not pro-inflammatory, suggesting that reduced ROS levels are insufficient to explain the CARS2 effect. Finally, reduced CARS2 levels did not alter the abundance of respiratory complexes or mitochondrial function. These results argue for an anti-inflammatory contribution of CARS2 in THP-1-derived macrophages, which extends beyond its canonical mitochondrial role.

## Introduction

The mitochondrial genome encodes its own unique set of tRNA species, requiring dedicated aminoacyl-tRNA synthetases (ARSs) to load their cognate amino acids. These 17 nucleus-encoded mt-synthetases (mt-ARSs) are responsible for attaching the correct amino acids to their specific tRNAs inside the mitochondrial matrix. Mt-ARS deficiency is associated with considerable clinical heterogeneity, which could reflect non-canonical roles for mt-ARS (1, 2). For instance, cysteinyl-tRNA synthetase 2 (CARS2) also functions as a cysteine persulfide synthase (CPERS), regulating mitochondrial biogenesis and bioenergetics (3).

By integrating expression data with biochemical analyses, we provided evidence that reduced *CARS2* expression predisposes to coronary artery disease (4). Macrophage *CARS2* suppression, unlike *MARS2* or *YARS2* knockdowns, was associated with increased expression and secretion of inflammatory cytokines. While the role of CARS2 in promoting IL-10 signaling was explored, the most strongly associated pathways were related to innate immunity, specifically interferon response. Interferons are primary activators of innate immunity, responsible for generic defense against pathogens (5, 6). Upon binding to cell surface receptors, interferons trigger a signaling cascade, resulting in increased expression of a group of effectors, the IFN-stimulated genes (ISGs). Classically, this involves the activation of the transcription factor STAT1 via post-translational modifications (7). The response can also be initiated intracellularly by the recognition of foreign nucleic acids by dedicated sensors (8, 9). The mitochondria play a central role in that regard (10–12). Reactive oxidative species (ROS) associated with mitochondrial dysfunction can also affect interferon signaling (13). In turn, interferon signaling may also increase ROS production in macrophages as a physiological response to infection (14).

Here, we continue our investigation into the anti-inflammatory contribution of CARS2, examining changes induced by reduced CARS2 abundance in THP-1 macrophages, a cell model widely used for functional investigations (15). Using standardized protocols involving phorbol ester treatment, THP-1 monocytes can be readily differentiated into macrophages (16). We confirm that sustained *CARS2* suppression results in transcriptomic changes consistent with increased interferon signaling in naïve THP-1 macrophages, although this was not accompanied by increased STAT1 phosphorylation. By contrast, *CARS2* suppression reduced NF-ΚB activity in an unpolarized THP-1 luciferase reporter model, despite evidence of increased inflammation. Interestingly, *CARS2* suppression resulted in seemingly normal respiration despite reduced ROS levels. Other parameters of mitochondrial function and integrity were unaffected by *CARS2* suppression. Together, these results demonstrate that reduced CARS2 abundance confers an atypical, inflamed environment in unpolarized macrophages, in the absence of detectable mitochondrial dysfunction.

## Material and methods

### Cell culture

THP-1 monocytes (ATCC, TIB-202) were cultured with glucose-free RPMI 1640 medium (Gibco) supplemented with 10% FBS, 2 mM L-glutamine, 100 U/mL penicillin and 100 μg/mL streptomycin, 10 mM HEPES, 5.5 mM glucose, 1.0 mM sodium pyruvate and 0.05 mM 2-mercaptoethanol. Cells were treated with 100 nM of phorbol 12-myristate 13-acetate (PMA) (Sigma Aldrich) for 72 h to differentiate into M0 macrophages. Silencing experiments were initiated immediately following M0 differentiation using 20 pM final siRNA and 1 ul of lipofectamine iMax per well (24-well plate).

### RelA luciferase assay

THP-1 NF-κBLuc-2 (TIB-202-NF-κB-LUC2, ATCC) human monocytes were cultured according to the manufacturer’s protocol. These cells harbor the firefly luciferase gene, under the control of the NF-κB promoter. NF-κB activation was measured using a luciferase assay. Cells were differentiated into M0 macrophages and treated with 100 ng/ml LPS or vehicle alone for 6 h. Alamar Blue reagent (DAL1025; Thermo Fisher Scientific) was added directly to the culture medium for 1 h. The fluorescence (ex/em (nm):355/596) was measured with a Cytation 5 Cell Imaging system.

30

Multimode Reader (BioTek). A Dual-Luciferase Reporter Assay System (E1910, Promega) was used according to the manufacturer’s protocol. Cells were lysed with 1X Passive Lysis buffer, and luminescence was measured on a GLOMAX Microplate Luminometer (Promega).

### Reverse transcription and quantitative real-time PCR

Total RNA was isolated from all samples using the High Pure RNA Isolation (Roche) and Direct-zol RNA (Zymo Research) kits, according to the manufacturer’s instructions. cDNA was generated using the Transcriptor First Strand cDNA Synthesis Kit (Roche), with 500 ng – 1 µg of RNA as the template. Quantitative PCR was performed using SYBR Green I Master Mix (Roche) and run on a LightCycler 480 Instrument II (Roche). Quantifications were done using Signal Recognition Particle 14 (*SRP14*) as the reference gene and the ΔΔCT method.

### Western blotting

Protein samples were resolved on an 8% or 12% SDS-PAGE gel. For the OXPHOS complex detection, samples were incubated at 37 °C for 30 min under reducing and denaturing conditions. For the other samples, the samples were denatured at 95 °C for 5 min in the same buffer. Nitrocellulose membranes were blocked for 1 h using Odyssey blocking buffer (Licor, 927-70001). The membranes were then incubated with primary antibodies in 5% Bovine Serum Albumin (BSA) in Tris-Buffered Saline with 0.1% Tween-20 overnight at 4 °C. IRDye-coupled donkey anti-rabbit or anti-mouse secondary antibodies (LI-COR) were used. Membranes were imaged using the LI-COR Odyssey imaging system. The membranes were probed for β-Tubulin as a loading control.

### Mitochondrial membrane potential (TMRE), mitochondrial content and ROS measurement

Mitochondrial membrane potential and content were measured using tetramethylrhodamine ethyl ester (TMRE) and MitoTracker Green, respectively. Cells were stained with 500 nM TMRE (Biotium) and 200 nM MitoTracker Green (Thermo Fisher, M7514) for 30 min at 37 °C in the dark. For ROS measurements, THP-1 macrophages were stained with 25 μM H2DCFDA (Thermo Fisher, D399) for 30 min in the dark at 37 °C. The media were then supplemented with 1 μM Hoechst for 5 min for normalization. Fluorescence of TMRE (ex/em (nm):550/580), MitoTracker Green (ex/em (nm):490 nm/516) Hoechst (ex/em (nm):350/461), and/or H2DCFDA (ex/em (nm): 495/529) on washed cells was measured using a microplate reader (BioTek). Blank (cell-free) well values were subtracted.

### Metabolic cell phenotype analysis

THP-1 monocytes (3.5x10^4^ cells/well) were plated on an Agilent Seahorse XFp Cell Culture Miniplate (Agilent) with 100 nM PMA to differentiate into macrophages. The Seahorse XF Base Medium (Agilent) was supplemented (4.5 g/L glucose, 2 mM L-glutamine, 1 mM sodium pyruvate), and the pH was adjusted to 7.4. Stock solutions of oligomycin A (100 μM), carbonyl cyanide p-trifluoromethoxy phenylhydrazone (FCCP) (100 μM), and rotenone/antimycin A (50 μM) from the Agilent Seahorse XF Cell Mito Stress Test Kit (Agilent Technologies) were diluted in the prepared medium. Hoechst 33342 dye (Thermo Fisher) was added to the medium (2 μL/mL) before addition to Port C (25 μL/port). Cells were counted using a Cytation 5 Cell Imaging Multimode Reader. The Agilent Seahorse XF Cell Mito Stress Test Report Generator was downloaded from Agilent to calculate baseline and stress phenotypes and the metabolic potential for each group. Data was analyzed with Wave 2.6.1 (Agilent Technologies).

### Pathway analysis

Transcript IDs (from the public GSE168863 dataset) that underwent > 1.5-fold change (p<0.05) were converted to Entrez ID (286 unique IDs) and subjected to enrichment analysis with Metascape (https://metascape.org/gp/index.html#/main/step1). Metascape performs over-representation analysis using several pathway and ontology datasets and subsequently clusters them according to membership similarity. Default parameters were used, except that COVID was removed; canonical pathways and Hallmark ontologies were included. GSEA was performed using WebGestalt (https://www.webgestalt.org/) with the default settings. Ingenuity Pathway analysis (IPA; Qiagen) was performed using the transcript IDs from HuGene-2_0-st to identify the top regulators.

### Statistical analysis

The results are presented as the mean ± standard deviation (SD) for independent experiments. Data points represent biological replicates. Significant differences were assessed by one-way ANOVA with significance set at p<0.05 (* p < 0.05, ** p < 0.01, *** p < 0.001, **** p < 0.0001) unless otherwise specified. Figures were produced and analyzed using GraphPad Prism 9.0.

## Results

### Clustering analysis of transcriptomic data reveals increased interferon activity in CARS2-suppressed cells

**Figure 1:**
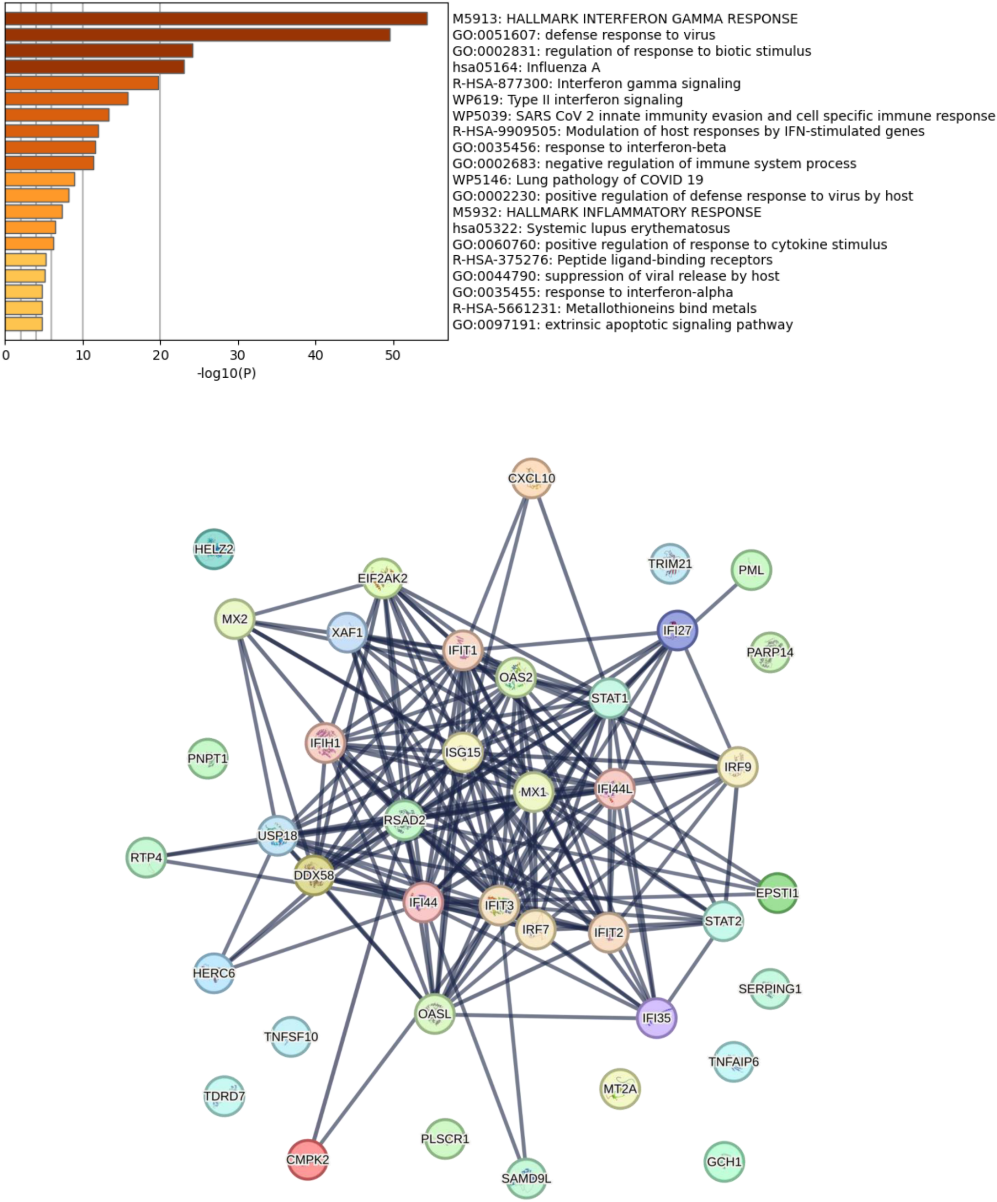
Prominent activation of innate immunity in CARS2-suppressed cells. Top, Metascape analysis of the most significantly impacted transcripts. Organized by clusters in decreasing statistical significance. The term shown is the most representative of each cluster. Bottom, string analysis of the 40 enriched genes populating the “Hallmark interferon gamma response”, showing extensive connectivity. Network edge thickness is proportional to confidence. Only highest confidence data was used to build the network.

We previously reported that gene set enrichment analysis (GSEA) of transcriptomic data from CARS2-suppressed M0 THP-1 macrophages identified enrichment for Reactome and Ontology terms related to innate immune response and interferon activation (4). To enrich and support these findings, the same dataset was subjected to overrepresentation analysis (ORA), focusing on the most perturbed transcripts. Metascape, which jointly performs over-representation and term clustering across distinct ontology families, was selected. The analysis revealed a strong enrichment of interferon-related terms, with “Hallmark Interferon gamma response” featuring prominently (**Figure 1****;** **Supplementary Table 1**). Unlike most ontology datasets, Hallmark gene sets are curated to inform on directionality (17). Examination of the underlying dataset revealed that the 40 genes that were mapped to that pathway were indeed upregulated, underscoring the authenticity of this enrichment. String analysis revealed extensive physical and functional interconnectivity among those genes, as expected.

To examine general trends in the dataset and to support the Hallmark ORA findings, GSEA was performed using the Hallmark gene sets. The analysis identified only 5 FDR-significant categories, including robust enrichment of interferon-related categories (Normalized effect size >6 and FDR < 2.2 E^-16^) and significant but less pronounced inflammatory and increased NF-κB responses (Normalized effect size =2.6 and FDR ~3 E^-4^) (**Supplementary Figure 1****).** Examination of the transcripts mapping the interferon alpha response gene set confirmed extensive upregulation (**Supplementary Table 2**).

### CARS2 suppression activates interferon signaling in the absence of significant STAT1 activation

We reasoned that the persistence of the inflammatory response (i.e., measured after 72 h of *CARS* suppression) might best align with activation of the STAT1 pathway, which mediates sustained inflammation (18). Interferons induce the transcription of interferon-stimulated genes (ISGs) via STAT1 activation, which could occur in a paracrine fashion (5). Moreover, the STAT1 transcript was increased in *CARS2-*suppressed cells and our previous upstream regulator analysis via Ingenuity Pathway Analysis (IPA) predicted increased STAT1 activation. The analysis was repeated to address dataset and pipeline updates, yielding similar findings: STAT1 was confirmed to be one of the most potent and likely transcription factors implicated (Corrected Z-score of 3.7 and Benjamini-Hochberg corrected p value of 3.9E^-29^) (**Supplementary Table 3**). Further support for STAT1 was obtained using GSEA analysis of transcription factor consensus sequences, demonstrating that upregulated genes were enriched in the ISRE1_01 motif, a STAT1/STAT2 binding site (**Supplementary Table 4**).

**Figure 2:**
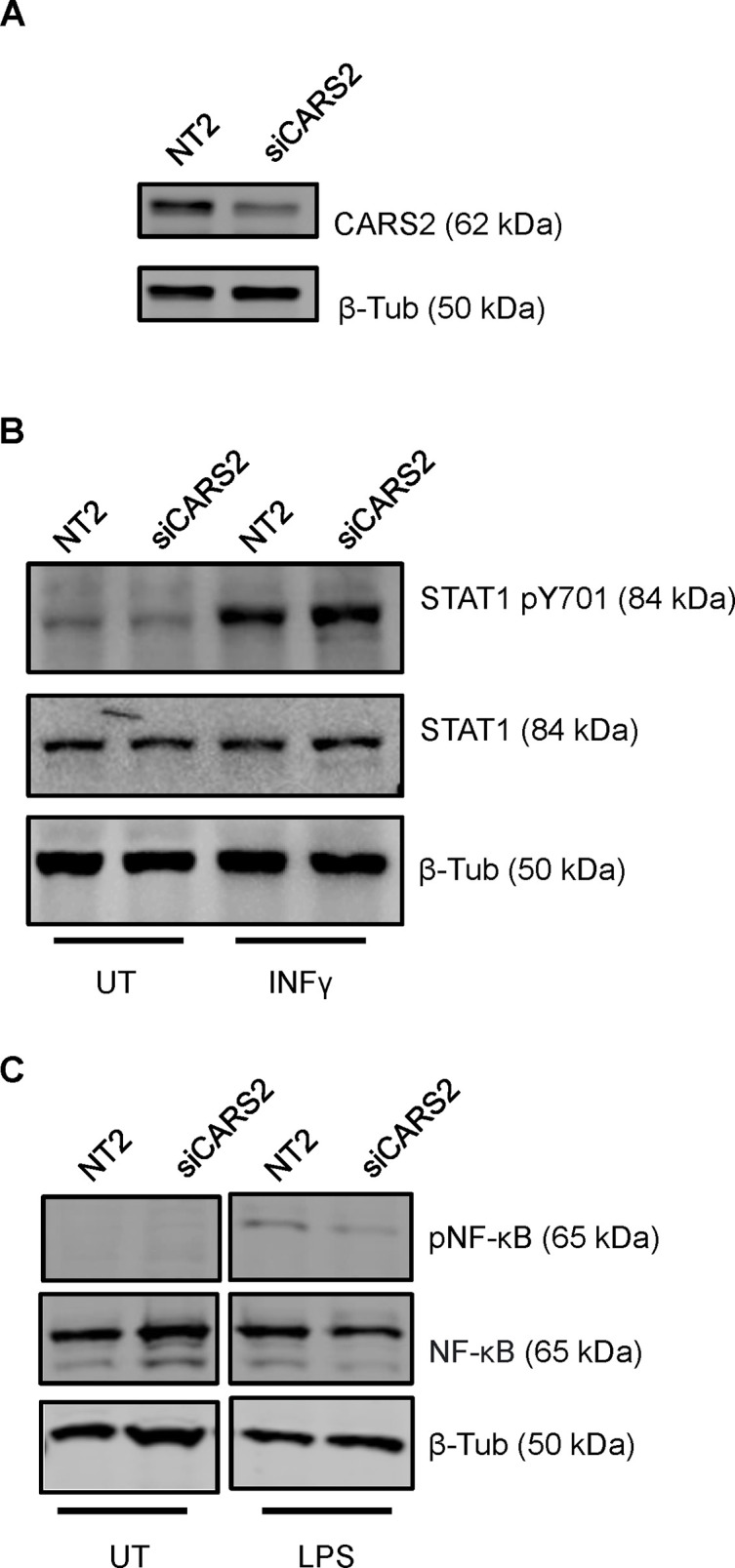
CARS2 suppression does not increase STAT1 or NF-κB (p65) phosphorylation. Western blot of siRNA-treated (72 h) THP-1 M0 cells, using the indicated antibodies. **(A)**, CARS2 abundance; **(B)**, STAT1 and pSTAT1 levels; **(C)**, NF-κB and pNF-κB levels. Cells were untreated (UT), treated with 20 ng/ml of IFNγ for 30 min or 15 min 100 ng/ml LPS as positive control. Representative of 3 independent experiments. Results are expressed relative to untreated or NT2 control siRNA values, as indicated. See **Supplementary Figure 2** for quantifications.

STAT1 is an important mediator of interferons: STAT1 phosphorylation (Tyr701) is crucial for initiating the response to interferon, whereas prolonged activation has been linked to increased STAT1 abundance in macrophages (18, 19). Western blot analysis was performed to measure STAT1 and STAT1 phosphorylation (pSTAT1) (**Figure 2**; see **Supplementary Figure 2** for quantification). Interestingly, *CARS2* suppression increased *STAT1* expression by 1.53-fold in the transcriptomic *CARS2* knockdown dataset (GSE168863), a change that was weakly correlated at the protein level (1.3-fold, p=0.12). In unstimulated cells, the pSTAT1 signal was faint, and not significantly different (relative to β-tubulin) between CARS2- and NT2si treated cells (**Figure 2B**). However, the relative pSTAT1/STAT1signal was reduced 1.3-fold (p=0.04) by *CARS2* suppression possibly reflecting increased STAT1 abundance. The addition of IFNγ elicited a rapid and potent stimulation of pSTAT1, that was not significantly augmented (1.6-fold, p=0.09) in CARS2-treated cells relative to non-target siRNA-treated cells. In summary, in unstimulated cells, *CARS2* suppression modestly reduced pSTAT1/STAT1, but not total STAT1 phosphorylation, suggesting a reconfiguration of the STAT1 pool without obvious activation.

### CARS2 suppression reduces NF-κB-dependent activity

**Figure 3:**
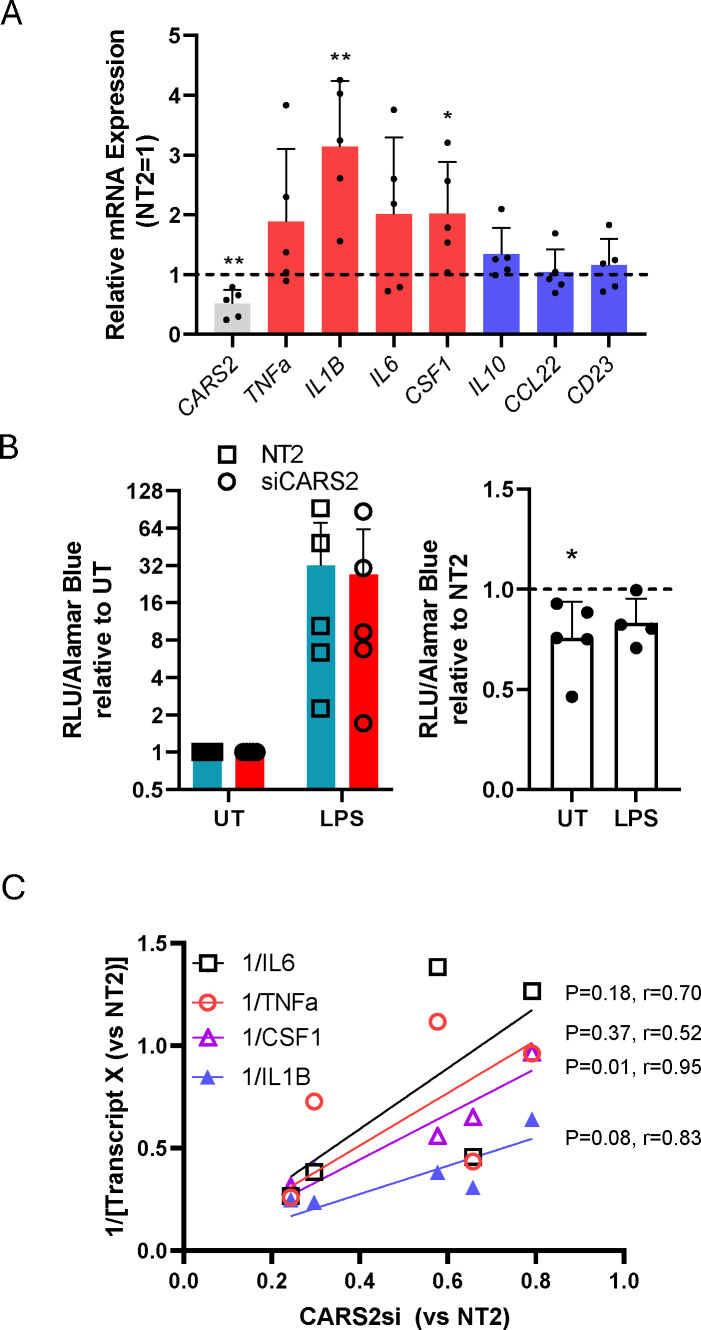
CARS2 suppression is inflammatory but is associated with reduced NF-κB activity. **(A)**, Reporter THP-1 M0 cells (TIB-202-NF-κB-LUC2) treated for 72 h with siRNA were analyzed for the expression pro- (red) and anti-inflammatory markers (blue) by qRT-PCR. **(B)**, Reporter THP-1 M0 cells were examined for NF-κB activity using a NF-κB responsive promoter luciferase assay. LPS (100 ng/ml was for 6 h) was used as a positive control. Results are expressed relative to untreated (UT) or NT2 control siRNA values, as indicated. **(C)**, Linear regression of inflammatory marker values in A. Reciprocal values were plotted as a function of the CARS2 values, with a x=0, y=0 constrain. Pearson correlation P and r values are indicated. *, p<0.05, **, p< 0.01.

In addition to interferon signaling, the Hallmark “TNFA signaling response mediated by NF-ΚB” pathway was identified by the GSEA and Metascape analyses. NF-κB is a major regulator of innate immunity, responsible for the rapid response to pathogens. Although this was inconsistent with our 72-hour time frame, we considered the possibility of delayed activation in response to cumulative stress. The NF-κB contribution was first assessed by western blot, using p65 phosphorylation levels as a proxy for activation (**Figure 2C****;** **Supplementary Figure 2** for quantification). In untreated cells, p65 phosphorylation was detected at very low levels, which stymied accurate quantification and potentially accounted for high variability. Predictably, inclusion of the NF-κB activator LPS strongly increased p65 phosphorylation, albeit in a CARS2-independent manner. To obtain a direct measure of NF-κB’s activity, *CARS2* suppression was repeated in a THP-1 reporter cell line stably transduced with a luciferase reporter driven by an NF-κB-responsive promoter. As previously reported for THP-1 macrophages, *CARS2* suppression in that cell line was associated with an inflammatory phenotype, characterized by increased expression of pro-inflammatory markers but little impact on anti-inflammatory markers (**Figure 3A**). Predictably, the addition of LPS strongly increased the promoter activity, consistent with NF-κB engagement (**Figure 3B**). Surprisingly, *CARS2* suppression reduced reporter activity, reaching nominal significance under basal conditions. Thus, despite exhibiting an inflammatory profile, *CARS2*-suppressed cells have reduced NF-κB activity.

### Inflammation extent is inversely proportional to *CARS2* suppression

We observed substantial variability in inflammatory marker expression in unstimulated reporter THP-1 macrophages, which we hypothesized may reflect the magnitude of *CARS2* suppression across experiments. Regression analysis revealed an inverse relationship between *CARS2* levels and inflammatory marker expression (**Figure 3C**). This linear association supports a causal link between *CARS2* suppression and the inflammatory phenotype, and suggests that small reductions in *CARS2* may be sufficient to enhance inflammation.

### *CARS2* suppression modulates responsiveness to inflammatory cues

To assess THP-1 macrophage responsiveness to inflammatory stimuli, expression of inflammatory cytokines was measured following LPS and IFNγ exposure. Addition of LPS increased expression of all transcripts, but this effect was different in *CARS2*-suppressed cells (**Supplementary Figure 3**). Whereas IL6 expression was stimulated at all doses and time points (although not reaching nominal significance) by the *CARS2* knockdown, *IL1B* was stimulated at low doses but inhibited at higher doses. Moreover, *CSF1* expression was stimulated at a higher LPS dose, whereas TNFA expression remained unchanged. By comparison, IFNγ exposure had little impact on our cytokine panel, with only *CSF1* showing increased expression, which was more pronounced in *CARS2*-suppressed cells. Overall, these cytokine-specific responses indicate that reduced CARS2 expression subtly reshapes the inflammatory responsiveness of THP-1 macrophages.

### Reduced radical oxygen species in CARS2-suppressed cells in the absence of salient mitochondrial defects

**Figure 4:**
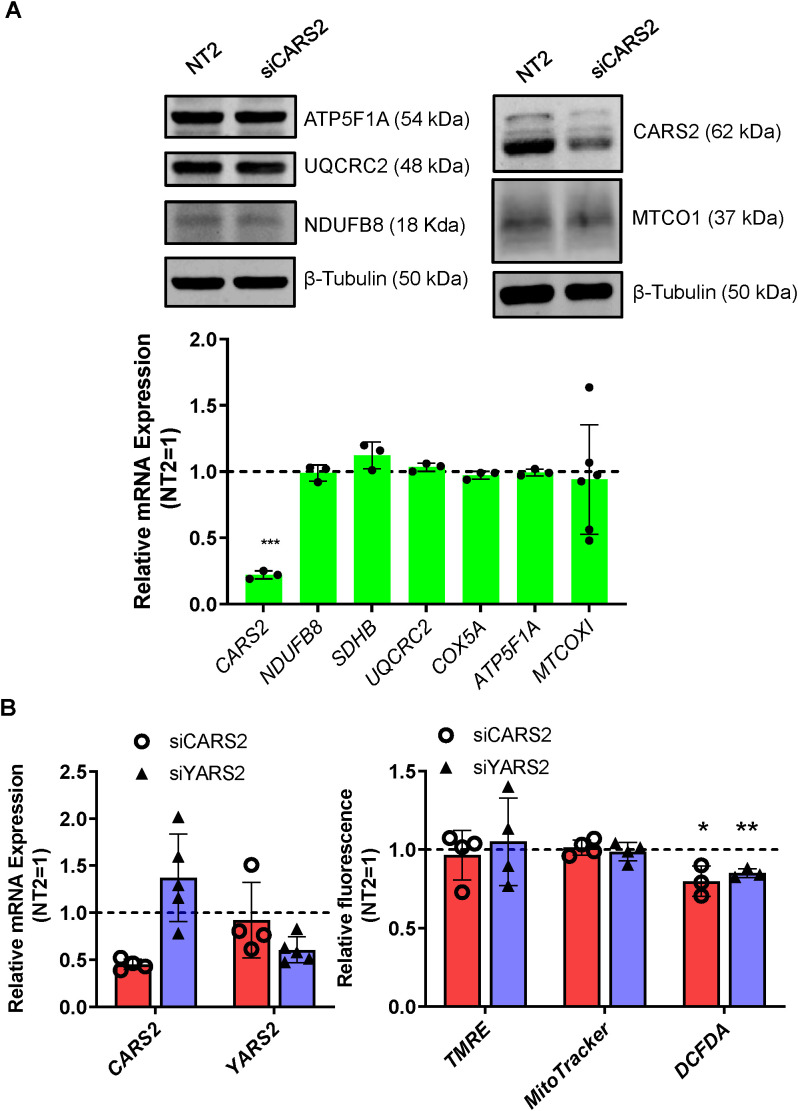
CARS2 reduces cellular ROS levels but do not affect mitochondrial membrane potential, mass or electron transport chain protein composition. Top, protein and RNA abundance as determined by western blotting and qRT-PCR. Bottom, impacts of CARS2 and YARS2 suppression on RNA abundance, Mitochondria membrane potential, ROS and mitochondrial content. Results are expressed relative to NT2 control siRNA values. *, p<0.05, **, p< 0.01.

Mitochondria are central to the innate immune response. CARS2 deficiency, elicited by profound *CARS2* suppression, was shown to have broad inhibitory effects on mitochondrial function, including membrane potential and oxygen consumption rate, and to lower the abundance of the mitochondrially-encoded mitochondrial cytochrome oxidase 1 (MTCO1) in HEK293T cells (3). By contrast, MTCO1 levels were normal in *CARS2*-suppressed THP-1 cells, possibly due to higher residual CARS2 levels (**Figure 4A**). Similarly, the abundance of other electron transport chain components was not affected. Moreover, there was no evidence of compensatory feedback affecting either nuclear or mitochondrially encoded transcripts.

Possible mitochondrial dysfunction was examined next. The tyrosine-specific mt-ARS YARS2 was concurrently suppressed, as we previously demonstrated that *YARS2* suppression was not pro-inflammatory, a finding we confirmed in an independent round of transfections (**Supplementary Figure 4**) (4). Fluorescence-based analyses did not detect changes in mitochondrial mass (MitoTracker) or membrane potential (TMRE) in mt-ARS-suppressed cells. However, unexpectedly, suppression was associated with reduced cellular ROS (**Figure 4B**). CARS2-deficient HEK293T exhibited a significant decrease in the oxygen consumption rate (OCR), indicating impaired respiration (3). By contrast, OCR (and the extracellular Acidification rate (ECAR), a measure of glycolysis) was unchanged in CARS2-suppressed THP-1 macrophages, under both basal and stressed conditions, arguing for preserved mitochondrial function (**Supplementary Figure 5**).

## Discussion

We previously reported to elicit increased inflammation, characterized by increased basal cytokine (TNF-α, IL-1β, IL-6, and M-CSF) secretion by *CARS2*-suppressed THP-1 macrophages, and the acquisition of pro-inflammatory and contractile-impaired profiles by smooth muscle cells in co-culture experiments (4). We argued that this inflammatory paracrine crosstalk between macrophages and smooth muscle cells may compromise vascular integrity, accounting for the genetically determined contribution of reduced CARS2 to CAD. Here, we have investigated the proximal effects of reduced *CARS2* expression, focusing on its intracellular impacts.

Clustering analysis of transcriptomic data from unstimulated cells predicted a modest, albeit statistically significant, activation of the canonical NF-κB pathway. By contrast, our previous GSEA analysis (confirmed in the present study) did not identify significant enrichment of the non-canonical NF-κB Gene Ontology (GO:0038061) and Reactome (R-HSA-5668541) categories, arguing that the slow-acting alternative pathway is not affected (20). Interestingly, a direct assessment of the canonical NF-κB activity via a dedicated reporter assay identified a slight decrease in NF-κB in *CARS2-*suppressed cells. Thus, the NF-κB ontologies used for transcriptomics mapping may not faithfully capture the specificities of THP-1 NF-κB signaling. Importantly, in the absence of stimulus, total activity was minimal, in line with very low NF-κB phosphorylation, regardless of CARS2 status.

By comparison, GSEA predicted clear activation of interferon signaling in *CARS2*-suppressed cells. Nevertheless, STAT1 phosphorylation was minimal, suggesting that STAT1 may not be implicated, at least not to a measurable extent. Indeed, although interferon signaling often converges on STAT1 (and other STATs), some aspects of interferon signaling persist in the absence of STAT1 (21). Moreover, the response in THP-1 cells may only share a subset of features with a bona fide interferon response. Indeed, naïve cells are not exposed to interferons, and it is doubtful that CARS2 suppression could fully recapitulate their signaling complexity. Thus, it seems more likely that reduced CARS2 alters the expression of genes associated with interferon signaling through mechanisms other than STAT1.

Another finding is that *CARS2* suppression had limited mitochondrial impact. Mitochondrial mass and membrane potential were seemingly normal, as measured by Mitotracker and TMRE assays. Judging from normal MTCO1 abundance, mitochondrial translation appeared largely intact, in line with previous work in Cars2+/- mouse livers, which display CARS2 reductions comparable to our siRNA interventions (3). Moreover, the Seahorse analysis did not detect differences in mitochondrial respiration (i.e., OCR), basally, and under stress (oligomycin + FCCP). Whether specific mitochondrial functional parameters (ATP-linked respiration, proton leak, or respiratory capacity) were altered remains unclear and would require using a Cell Mito Stress Test.

Although ROS levels were slightly reduced, this decrease may be due to changes in alternative ROS sources, as they are also generated by non-mitochondrial processes. However, as *YARS2* suppression also reduced ROS, an impact via the mitochondria seems likely. Unfortunately, attempts to measure mitochondria-generated ROS (using MitoSox) in THP-1 were unsuccessful due to insufficient signal. It is unclear how reduced mitochondrial ROS coexists with normal mitochondrial function. Nevertheless, our results suggest that changes in ROS levels may not be linked to the inflammation phenotype for two reasons. For one, *YARS2* suppression also reduced ROS levels without evident inflammation. In addition, inflammation is typically associated with increased ROS (22).

Although this work clarifies the pathways involved and exonerates obvert mitochondrial dysfunction, it does not yet explain how CARS2 suppression drives inflammation at the cellular level. One possibility is through reduced CARS2 CPERS activity, which is a major source of cellular persulfides needed for protein sulfidation (3). Indeed, persulfides have been shown to downregulate innate immunity in macrophages, suggesting that lower CARS2 abundance may lead to persulfide insufficiency and a pro-inflammatory state (23, 24). This will be the focus of future investigations.

## Data Availability

The datasets presented in this study can be found in online repositories. The names of the repository/repositories and accession number(s) can be found below: https://www.ncbi.nlm.nih.gov/geo/, GSE168863.
