# Supplementary figures and images for: Reduced CARS2 expression elicits a low-grade pro-inflammatory signature in THP-1 macrophages
Source: Front Immunol. 2026 Jun 5;17:1786365. doi: 10.3389/fimmu.2026.1786365 (PMC13278915; doi:10.3389/fimmu.2026.1786365)

STAT1

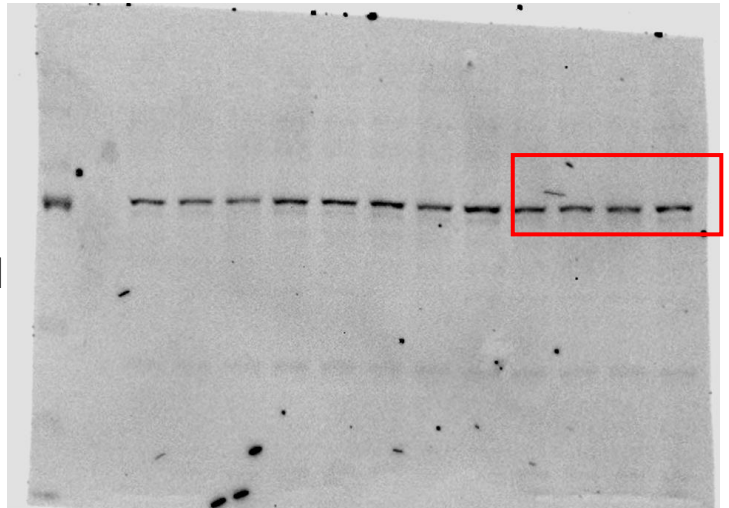

PSTAT1

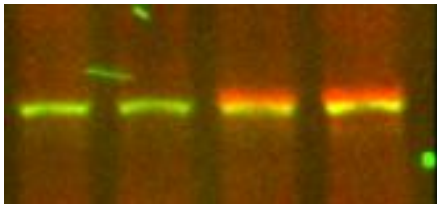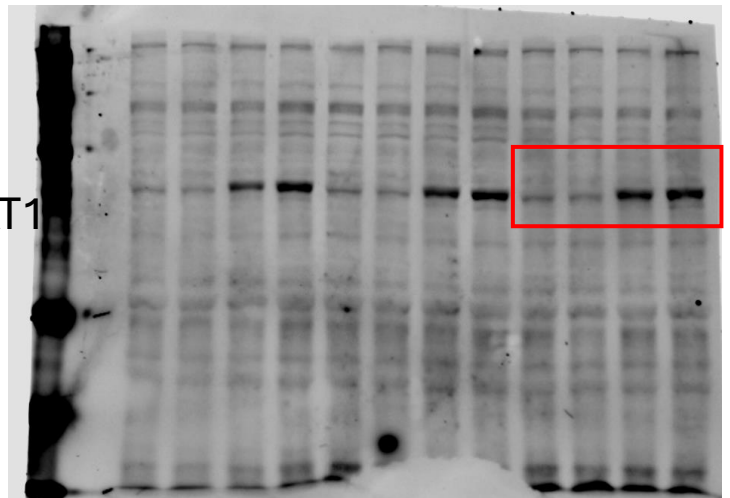

TUBB

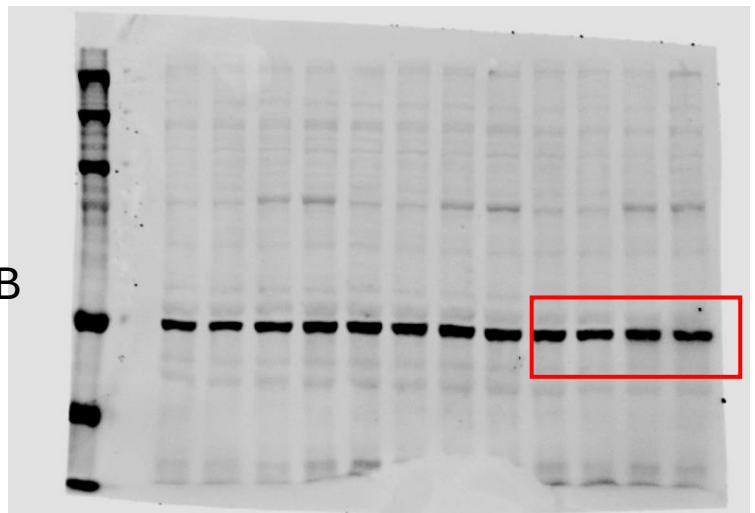

cars2

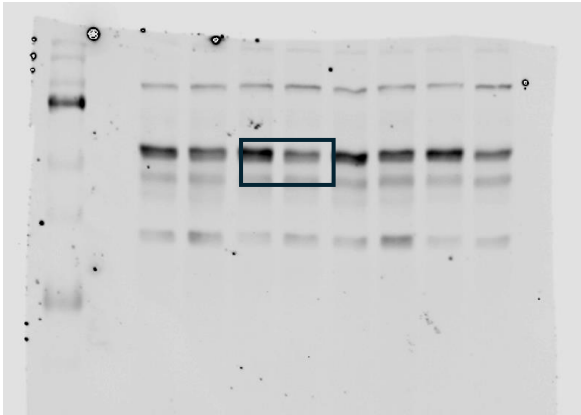

tubb

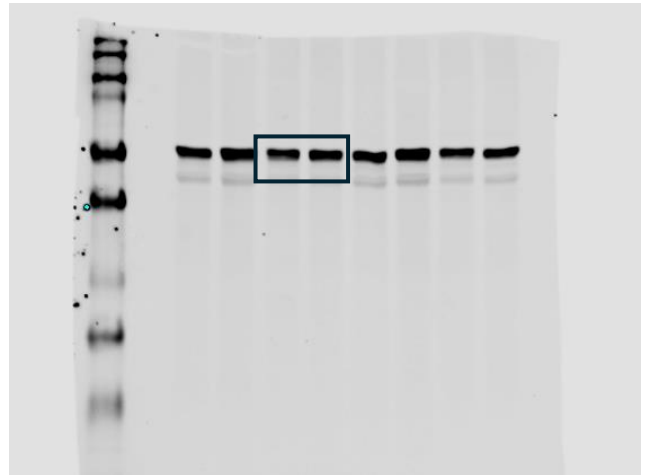

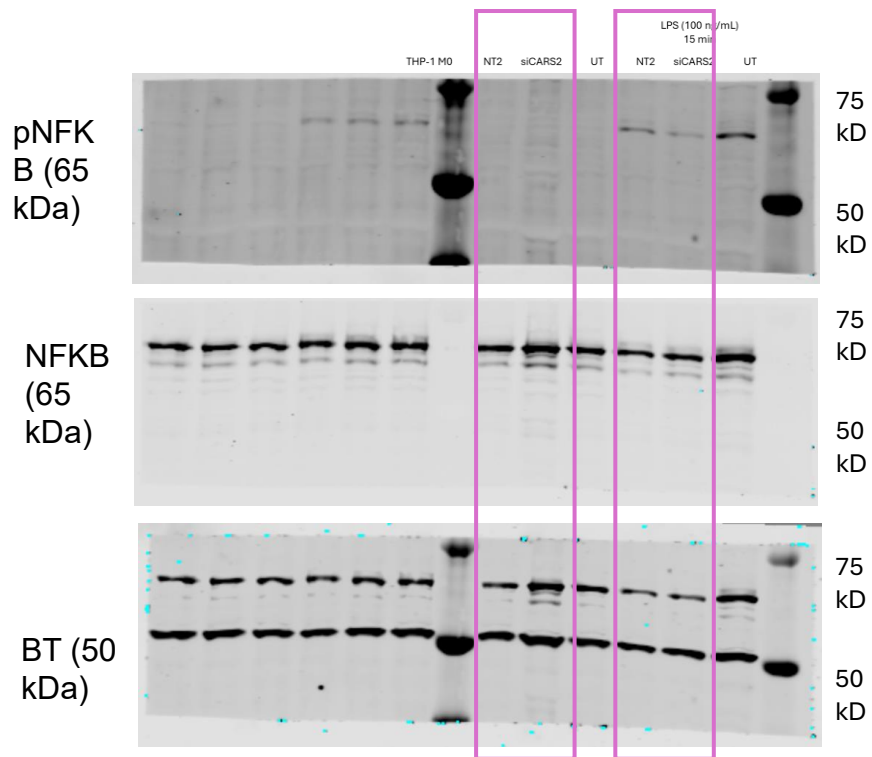

V - ATP5A (54 kDa)  
III - UQCRC2 (48 kDa)  
I-NDUFB8

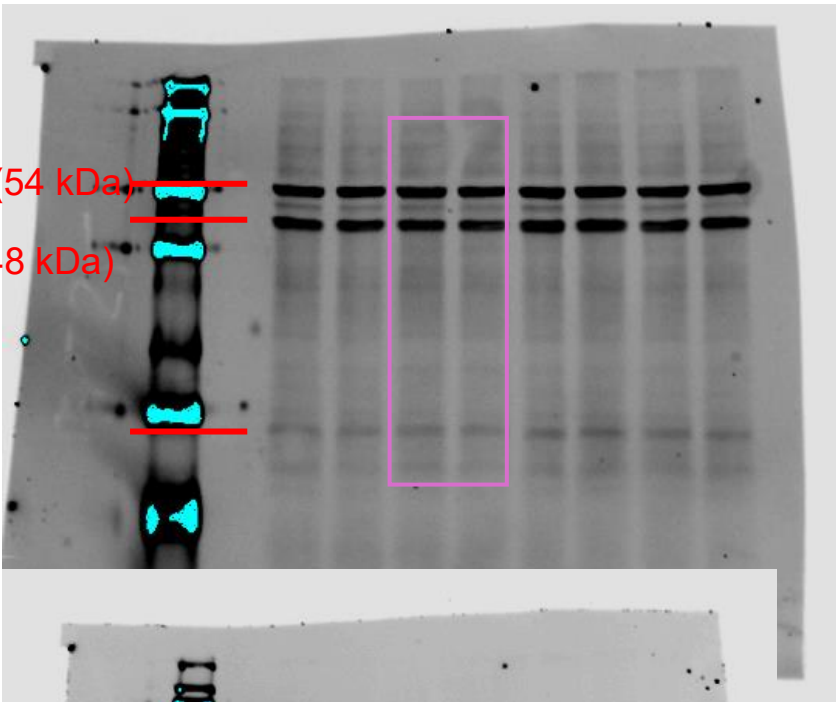

tubb

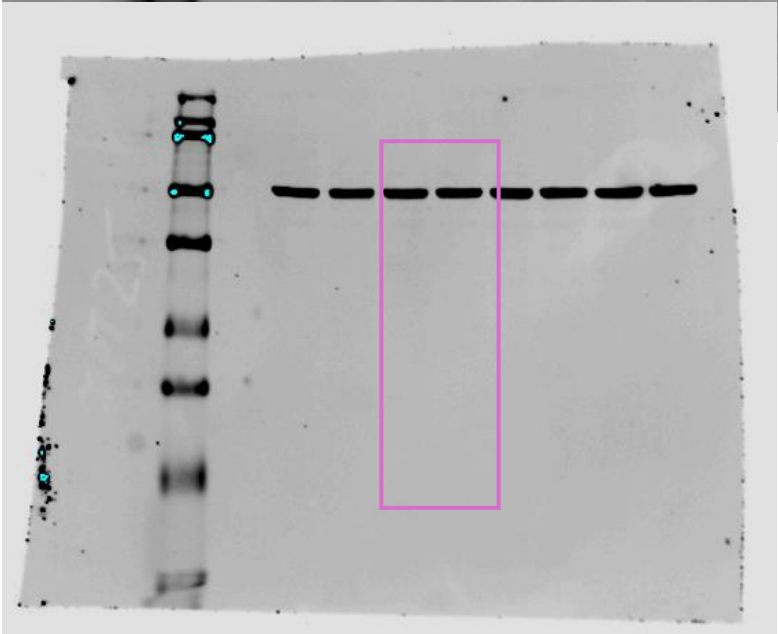

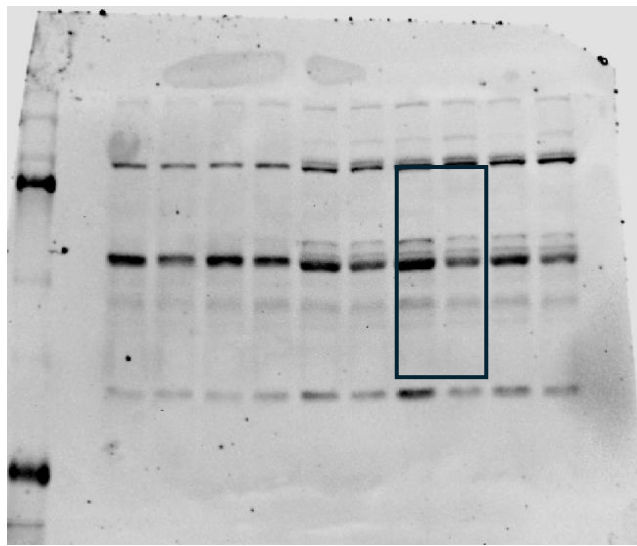

CARS2

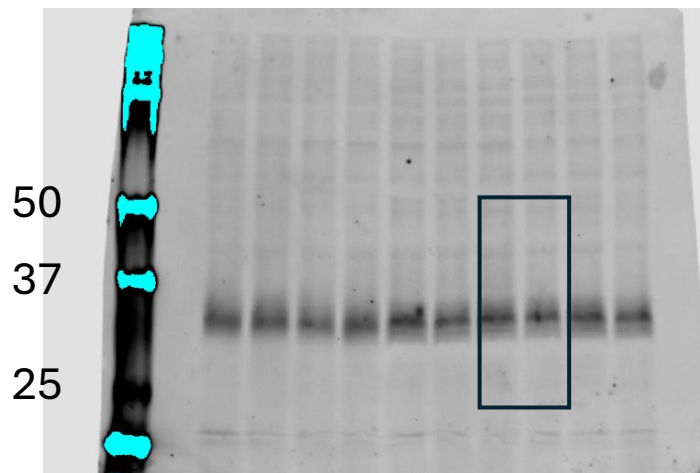

MTCO1

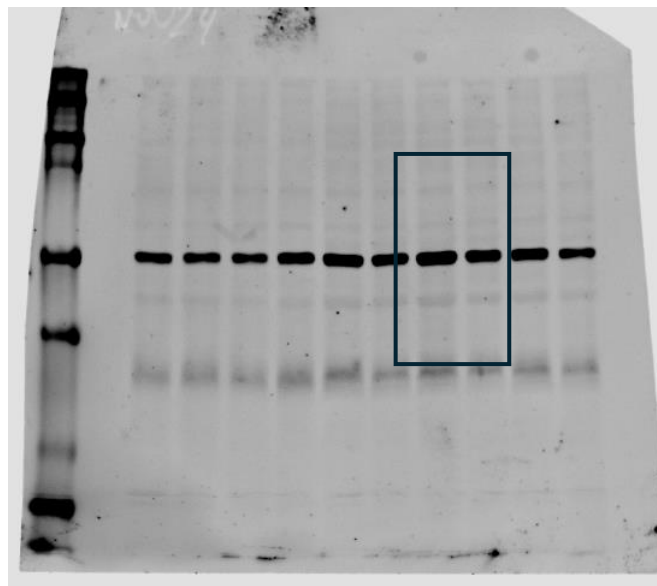

TUBB

Supplement: Supplementary file 1 [file DataSheet1.pdf]
