# Supplementary material for: Reduced CARS2 expression elicits a low-grade pro-inflammatory signature in THP-1 macrophages
Source: Front Immunol. 2026 Jun 5;17:1786365. doi: 10.3389/fimmu.2026.1786365 (PMC13278915; doi:10.3389/fimmu.2026.1786365)
Supplement: Supplementary file 3 [file SupplementaryFile2.pdf]

Gene Set	Size	L.E.N.	ES	NES	P Value	FDR
HALLMARK_INTERFERON_ALPHA_RESPONSE	93	63	0,68	6,78	<2.2e-16	<2.2e-16
HALLMARK_INTERFERON_GAMMA_RESPONSE	191	91	0,51	6,72	<2.2e-16	<2.2e-16
HALLMARK_TNFA_SIGNALING_VIA_NFKB	194	74	0,20	2,63	<2.2e-16	2,6E-04
HALLMARK_INFLAMMATORY_RESPONSE	198	76	0,20	2,65	<2.2e-16	3,5E-04
HALLMARK_COMPLEMENT	190	110	0,15	1,99	5,8E-03	2,0E-02

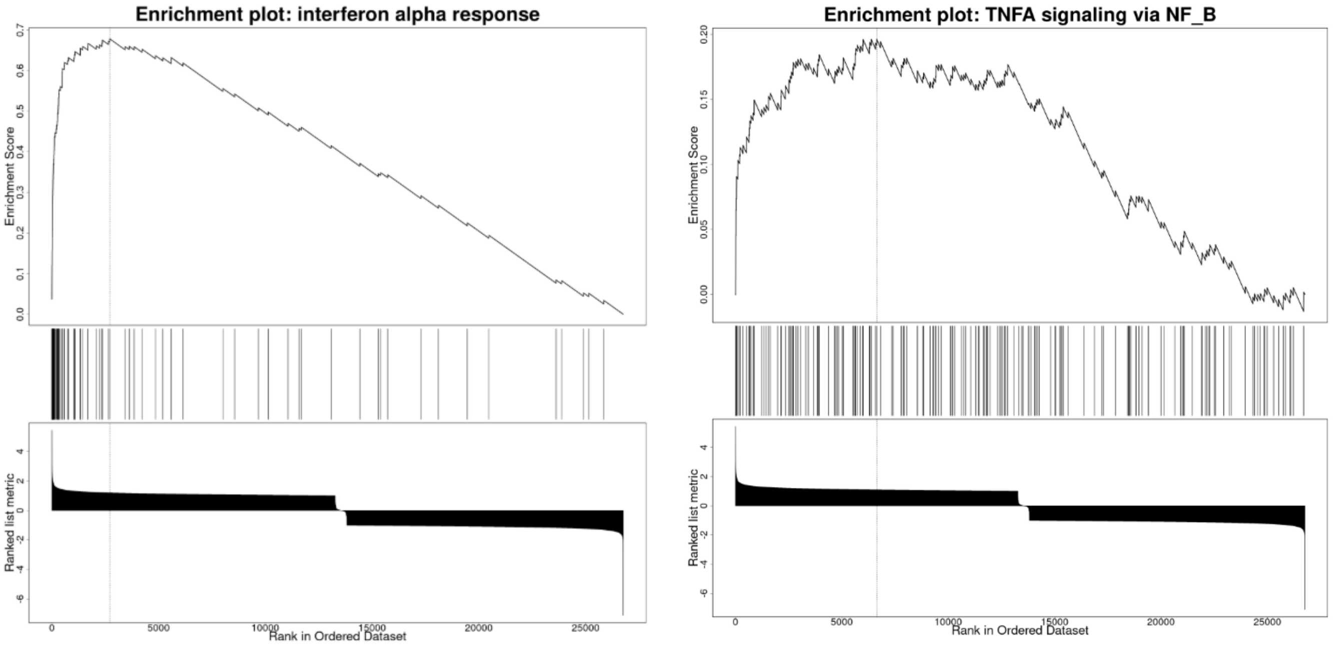

Figure S1. FDR-significant Hallmark categories identified by gene set enrichment analysis of CARS2-suppressed cells. Top, table of FDR significant hits**.** Size: members of the category present in the transcriptomic dataset; L.E.N. leading edge number; ES, effect size; NES, Normalized effect size; P Value, nominal p value of the enrichment; FDR, false-discovery q value of the enrichment. Bottom, enrichment plots of “HALLMARK_INTERFERON_ALPHA_RESPONSE” and “HALLMARK_TNFA_SIGNALING_VIA_NFKB” sets illustrating the strong enrichment of the interferon alpha related transcripts.

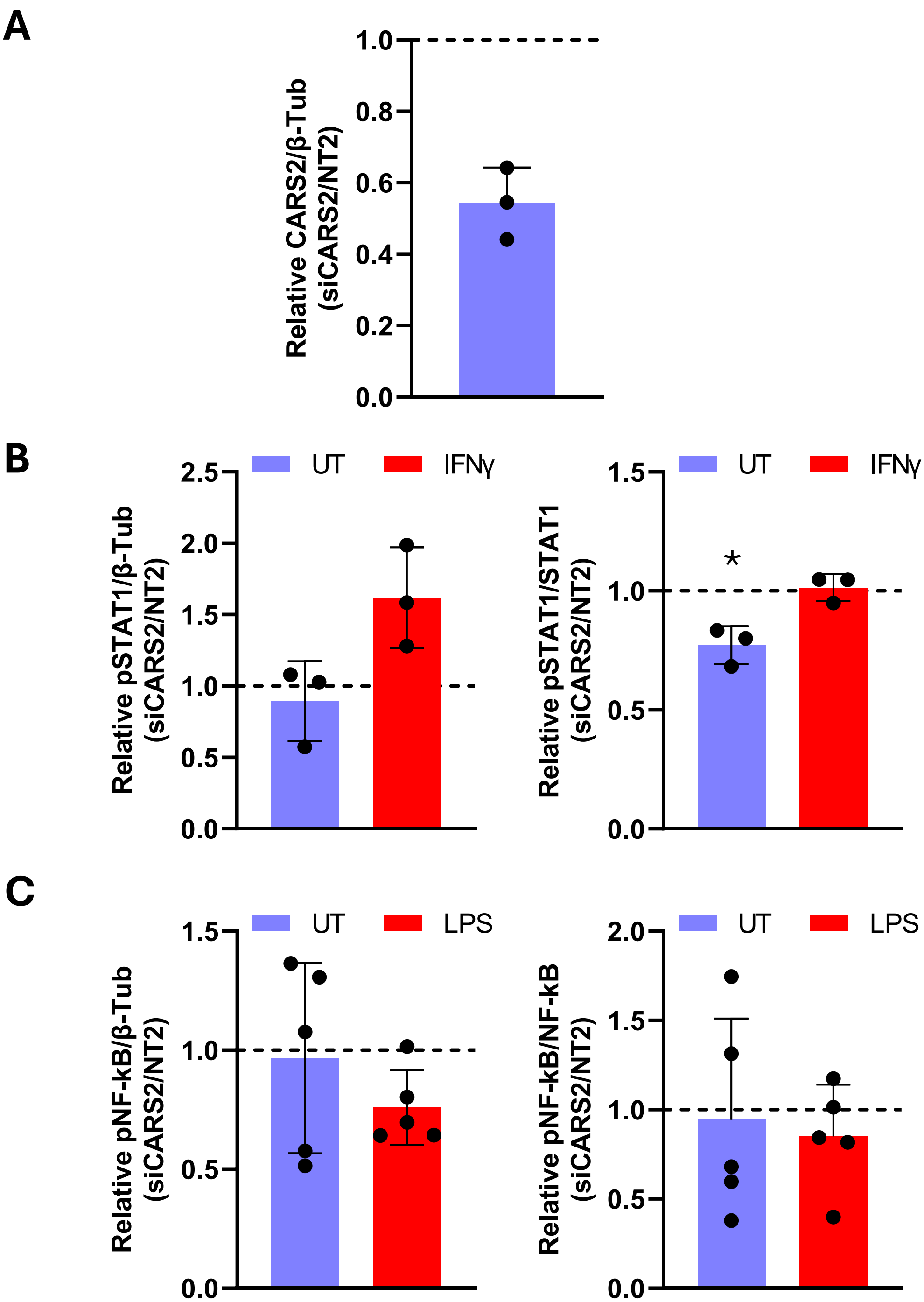

Figure S2. Western blot quantification. A, B and C represent the western blot quantification of Figure 2A, B and C. Western blot signals were first normalized to β-Tub or matching protein signal, as indicated, and data is expressed as the CARS2si sample values over the NT2si values.

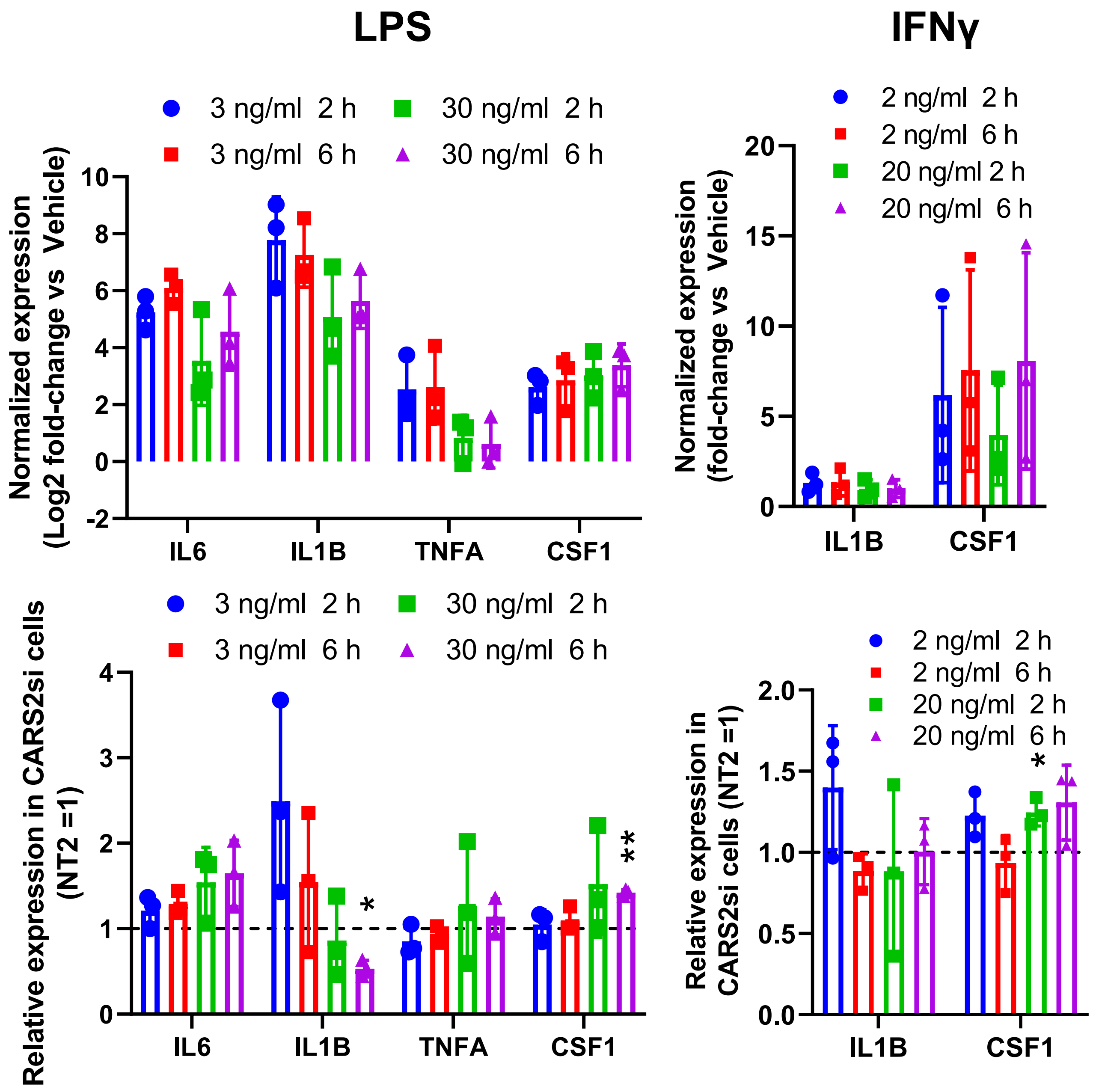

Figure S3. Inflammatory profile in CARS2-suppressed cells in response to LPS and IFNg. qRT-PCR quantification of cytokines in CARS2si-suppressed cells (72 h). Cells were exposed to LPS (3 or 30 ng/ml) or IFNγ (2 or 20 ng/ml) for the indicated time. IL6 and TNFA could not be confidently measured in the IFNγ experiments. Top, log_2-fold-change of transcript abundance; only the NT2 values are shown for simplicity. Bottom, ratio of CARS2si to NT2 values. Data points represent biological replicates (n=3), and error bars are SD. Statistical significance was determined using a t-test vs a theoretical value of 1.

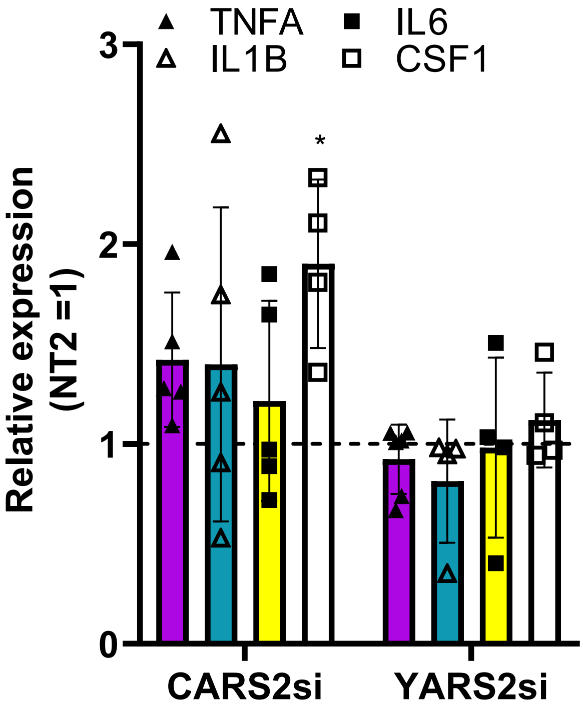

Figure S4. Pro-inflammatory profile in CARS2-suppressed cells. qRT-PCR quantification of inflammatory markers in CARS2si- and YARS2si-suppressed cells (72 h). Experiments were performed in parallel.

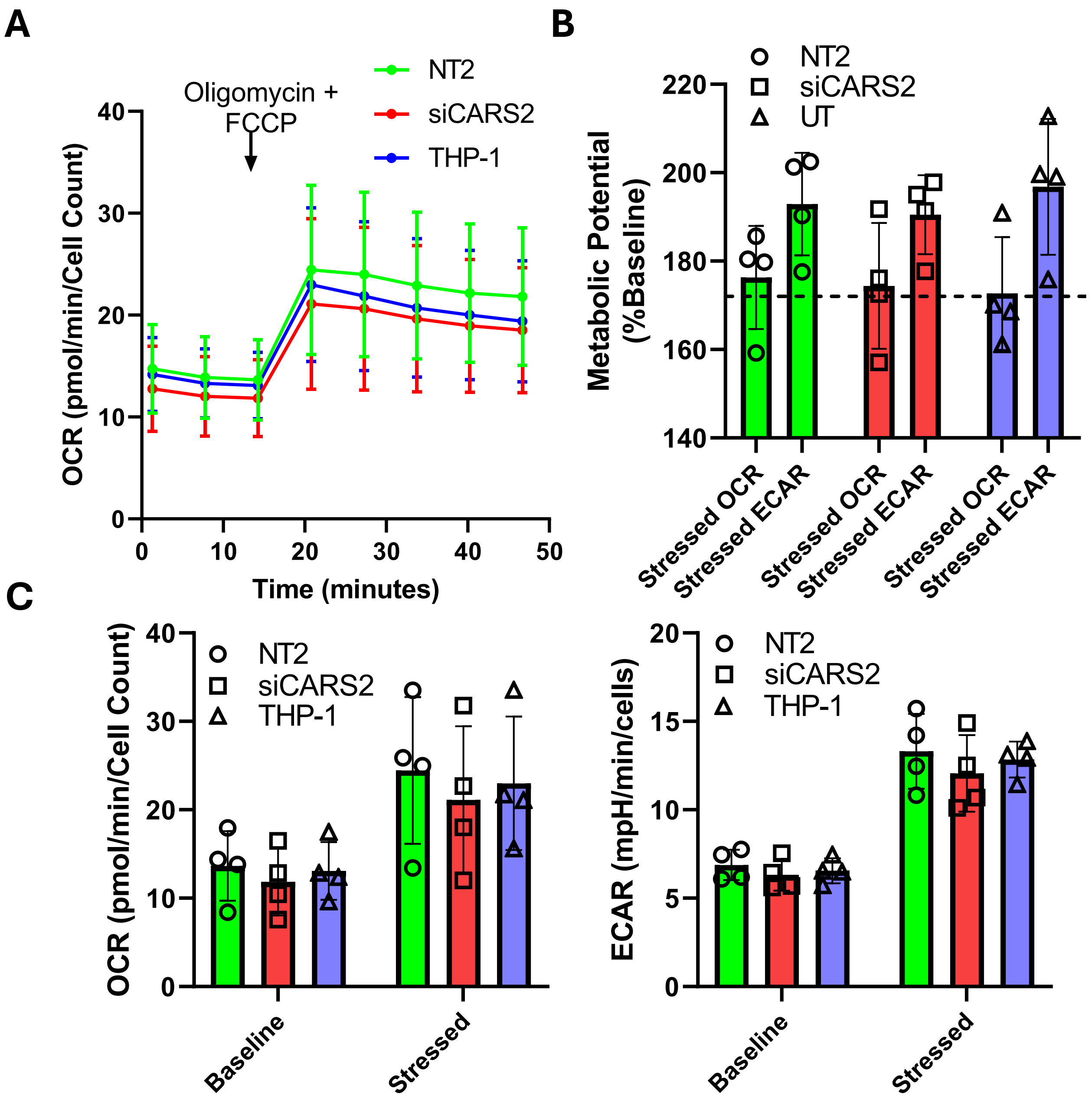

Figure S5. Metabolic potential in THP-1 macrophages is unaffected by CARS2 suppression. THP-1 macrophages were either transfected with *CARS2si, a* control siRNA (NT2), or left untreated (UT) for 72 h. The metabolic profile was determined using a Seahorse XF Cell Energy Phenotype assay. The oxygen consumption rate (OCR) and extracellular acidification rate (ECAR) were measured at baseline and under stress conditions (addition of 1 μM Oligomycin A and 0.5 μM FCCP). **(A)** OCR levels versus time at baseline and under stress conditions. **(B)** Metabolic Potential is expressed as the percent change in OCR and ECAR under stress relative to baseline conditions. (**C**) Summary data for OCR and ECAR. Data represent the mean ± SD for n=4.
